# Comparative modeling of mixed cardiopulmonary sounds in a low-resource paired dataset: Discrimination, calibration, and operating-point behavior

**DOI:** 10.1371/journal.pone.0352180

**Published:** 2026-06-22

**Authors:** Runchen Cai

**Affiliations:** Faculty of Mathematics and Computer Science, Quanzhou Normal University, Quanzhou, China; Universiti Sains Malaysia, MALAYSIA

## Abstract

**Background:**

Mixed cardiopulmonary recordings are common in bedside auscultation, yet most automated systems have been developed for isolated heart sounds or isolated respiratory sounds.

**Methods:**

We conducted a comparative methods study on HLS-CMDS, a low-resource paired dataset containing mixed recordings with matched isolated heart and lung source recordings. The task was dual binary classification from a single mixed recording. We compared feature-based references, a shared-backbone multitask CNN, a target-domain student model, teacher-guided variants pretrained on PhysioNet/CinC 2016 and ICBHI 2017, and lighter source-aware variants using paired HLS-CMDS source recordings. A nested grouped five-fold evaluation was performed at the triplet level; within each outer training fold, an inner validation split was used for checkpoint selection, temperature scaling, and task-specific threshold selection.

**Results:**

Under the revised nested evaluation, the light source-aware model showed the strongest mean discrimination (macro AUROC 0.7107 ± 0.1659; macro AUPRC 0.9318 ± 0.0423). The prevalence-defined no-skill macro AUPRC baseline was 0.8586 ± 0.0225. After inner-validation temperature scaling and threshold selection, the calibrated student-only model achieved the highest mean macro balanced accuracy (0.6894 ± 0.0548). The observed differences were interpreted cautiously because fold-to-fold variability was substantial.

**Conclusions:**

In this small paired mixed-sound setting, restrained source-aware guidance showed the strongest discrimination tendency, whereas a simpler target-domain model achieved the best threshold-dependent balanced accuracy after calibration.

## 1 Introduction

Automated analysis of physiological sounds has progressed rapidly for isolated phonocardiograms and isolated respiratory recordings, yet the mixed case—the one closest to routine bedside listening—remains comparatively underexplored. In everyday auscultation, cardiac and pulmonary components overlap in time and frequency, so methods developed for a single source do not necessarily transfer intact to mixed recordings.

That overlap complicates more than feature extraction. It changes how performance should be judged. In preliminary experiments, several models separated positive and negative recordings reasonably well on ranking metrics while collapsing into one-sided operating behavior once a threshold was applied. For a task meant to support binary decisions, that distinction matters.

HLS-CMDS provides an unusually useful setting for examining this problem because the mixed subset is paired with corresponding isolated heart and lung recordings. This structure makes it possible to compare plain target-domain learners, multitask baselines, teacher-guided models, and source-aware formulations under the same target-domain constraints. The target dataset and the two external teacher corpora used in this study are public resources [1–3].

Prior literature offers strong starting points but not a settled recipe for this setting. Competitive results have been reported for CNN-based heart-sound classifiers, multitask respiratory-sound models, and teacher-guided acoustic networks, but most of that work addresses single-source tasks or recording conditions that differ materially from paired mixed auscultation. Heart–lung separation studies show that the two sources can be disentangled, yet separation is not the only reasonable strategy when paired source recordings are already available. These prior streams include heart-sound classification, respiratory-sound classification, multitask learning, and related teacher-guided acoustic work [4–13]; separation-based studies address a different but relevant route to the mixed-source problem [14–16].

We therefore treated the present study as a comparative methods paper rather than an architecture paper. The central questions were practical. How far can a simple target-domain learner go on a small paired mixed-sound corpus? Does external supervision from large single-source datasets help once the target domain is genuinely mixed? And does paired source information improve discrimination once operating-point behavior is evaluated explicitly?

The aim was not to argue that one model family is universally superior. Rather, we sought to identify which modeling choices remain useful once the task is evaluated on a small paired mixed-sound dataset and judged with both discrimination and decision-oriented summaries.

**Fig 1:**
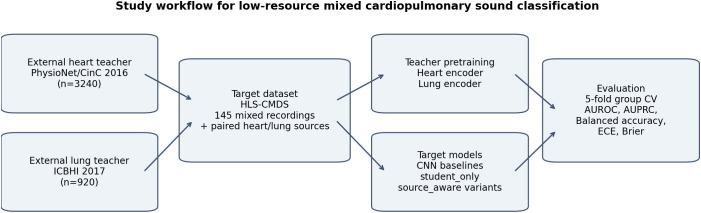
Study workflow, dataset flow, comparative model families, and evaluation endpoints.

Fig 1 summarizes the study workflow, dataset flow, comparative model families, and evaluation endpoints.

## 2 Materials and methods

### 2.1 Study design and reporting framework

This work was designed as a comparative prediction-model study for mixed cardiopulmonary sound classification. The target task was dual binary classification from a single mixed recording: one label for the cardiac component and one for the pulmonary component. The analysis followed the logic of a benchmark-style methods comparison, with explicit separation between ranking performance and thresholded decision behavior.

The principal target-domain analysis used nested grouped five-fold cross-validation over 145 paired triplets. Each triplet served as one grouping unit and contained one mixed recording together with its matched isolated heart and lung recordings. The original outer folds were preserved at the triplet level so that matched recordings could not be split across folds. Within each outer training fold, a group-disjoint inner validation split was used for checkpoint selection, calibration, and threshold selection. The outer fold was reserved for final evaluation only.

### 2.2 Datasets

Three public datasets were used. HLS-CMDS served as the target-domain dataset [1]. After metadata harmonization and manual audit, the processed HLS index contained 536 rows, of which 535 analyzable recordings formed the study corpus and one additional example was retained for audit only; the final comparative analysis used 145 complete mixed-recording triplets. No new human-subject data were collected for the present study.

PhysioNet/CinC 2016 was used as the external cardiac teacher corpus [2]. After preprocessing, 3240 heart-sound recordings were indexed for teacher training. ICBHI 2017 was used as the external pulmonary teacher corpus [3], yielding 920 indexed respiratory recordings after preprocessing. Healthy recordings were treated as the normal class and all diagnosed respiratory conditions were grouped as abnormal for pulmonary teacher pretraining. The purpose of using these corpora was not to claim direct clinical equivalence across datasets, but to inject organ-specific prior knowledge into the target-domain learner.

### 2.3 Signal processing and input representation

All audio pipelines were standardized to mono 4-kHz processing in the final configuration. Neural models received 64-bin log-Mel spectrograms generated from 15-s windows using a 512-point short-time Fourier transform and a 160-sample hop. This representation was chosen to harmonize the target-domain and teacher-domain pipelines while keeping the computational burden modest relative to the small target corpus. For hand-crafted baselines, mixed-recording descriptors included MFCC summary statistics, delta and delta-delta terms, spectral centroid, bandwidth, roll-off, zero-crossing rate, and energy-related descriptors.

For the feature-based reference path, mixed-recording descriptors were derived from MFCC statistics, delta and delta-delta terms, spectral centroid, bandwidth, roll-off, zero-crossing rate, and energy summaries. These hand-crafted features were retained to verify that the target task contained learnable structure even under simple representations. However, the principal manuscript conclusions were based on the neural models because the complete five-fold campaign was performed for those systems.

### 2.4 Target tasks and labels

Each mixed recording was associated with two binary labels. The heart label denoted whether the cardiac source was abnormal, and the lung label denoted whether the pulmonary source was abnormal. This dual-task formulation was selected for two reasons. First, it respects the fact that mixed recordings may simultaneously contain a normal signal from one organ and an abnormal signal from the other. Second, it creates a natural test bed for shared-representation learning, because both outputs are derived from the same acoustic input.

The main evaluation set therefore consisted of 145 paired triplets. Each outer test fold contained approximately 29 mixed recordings, while the corresponding outer training portion was further divided into inner training and inner validation subsets. With partitions of that size, noticeable fold-to-fold fluctuation was expected. Accordingly, the manuscript treats the five-fold means, standard deviations, and fold-level supplementary results as descriptive comparative evidence, whereas single-fold examples are used only to illustrate specific behaviors.

### 2.5 Model families

Four model families were studied. The first was a shared-backbone CNN baseline. It used one acoustic trunk followed by two task-specific output heads, thereby testing whether straightforward multitask learning improved over separate single-task CNNs. The second was the student-only mixed-sound model. This model also operated directly on the mixed recording, but it was kept free of external teacher constraints and source-aware regularization. In effect, it represented the strongest pure target-domain learner. The CNN baselines and heart-sound modeling choices follow common log-Mel/CNN and benchmark practices [17–21].

The third family introduced dual teachers. A heart teacher was pretrained on PhysioNet/CinC 2016 and a lung teacher on ICBHI 2017. Their outputs regularized the student through auxiliary losses. Two source-aware variants additionally used paired isolated heart and lung recordings from HLS-CMDS to constrain the mixed-recording representation. In practice, the most informative comparison was not simply teacher versus no teacher, but heavy versus light auxiliary weighting. The final candidate that emerged from this progression was a lightly regularized source-aware model.

Teacher checkpoints were trained independently before target-domain comparison. On the fold-0 teacher validation sets, the PhysioNet teacher reached AUROC 0.9572, whereas the ICBHI teacher reached AUROC 0.9855. The lung teacher, however, showed pronounced threshold bias at the default operating point. These fold-0 teacher summaries were used as adequacy checks before target-domain comparison rather than as stand-alone claims about transfer performance.

### 2.6 Calibration, threshold selection, and metrics

Ranking metrics alone were considered insufficient because early validation experiments revealed marked threshold sensitivity. The revised evaluation therefore separated discrimination, calibration, and threshold-dependent behavior more explicitly. Macro AUROC was used as the primary discrimination endpoint, with macro AUPRC as a complementary ranking metric. Because both target labels were highly prevalent in the mixed subset, AUPRC values were interpreted alongside prevalence-defined no-skill AUPRC baselines.

Temperature scaling and threshold selection were performed within each outer training fold only. Specifically, each outer training portion was split into inner training and inner validation subsets. Models were trained on the inner training data, and the inner validation data were used for checkpoint selection, temperature scaling, and separate heart and lung threshold selection. Temperature parameters were estimated by minimizing negative log likelihood on the inner validation predictions. Heart and lung thresholds were then selected on the calibrated inner validation probabilities to maximize balanced accuracy. The selected temperatures and thresholds were applied once to the untouched outer fold for final evaluation. This same calibration and threshold-selection pipeline was applied to all principal neural models. Temperature scaling and calibration summaries followed established neural-network and clinical-prediction calibration practice [22,23].

### 2.7 Statistical analysis, reproducibility, and final comparison set

The principal comparisons focused on four calibrated final candidates: the student-only model, the shared-dual CNN, the light dual-teacher model, and the light source-aware model. All four models were evaluated under the same nested grouped five-fold procedure. Because only five grouped outer folds were available and fold-wise estimates are not statistically independent replicates, the cross-validation summaries were treated as descriptive comparative evidence rather than as a basis for formal significance testing. We therefore report fold-wise means, standard deviations, and model contrasts, and we judge consistency across endpoints together with absolute performance.

All analyses were implemented in Python using openly available scientific-computing libraries. The archived supporting package includes the finalized parameter settings, fold-definition tables needed to reconstruct the evaluation, per-fold summary metrics, out-of-fold predictions, and the scripts used to generate the reported analyses. The revised reporting package was also checked against TRIPOD+AI and STARD-AI guidance, and a completed STARD-AI checklist is supplied as Supporting Information [24,25].

## 3 Results

### 3.1 Target-dataset profile

The curated HLS-CMDS metadata table confirmed that the supervised mixed-task subset was structurally clean and suitable for paired evaluation. All 145 identifiers corresponded to complete triplets composed of one mixed recording together with its matched isolated heart and isolated lung source recordings. The mixed subset was strongly imbalanced: 91.0% of recordings were heart-abnormal and 80.7% were lung-abnormal. Such imbalance made it unsurprising that some early models achieved high sensitivity while collapsing specificity at the default operating point.

The location distribution was relatively broad rather than dominated by a single site. Right costal margin (RC), right upper sternal border (RUSB), left costal margin (LC), and left mid anterior (LMA) each contributed at least 13 mixed recordings, while the least frequent location, right upper anterior (RUA), still contributed 9. This spread is modest, but it reduces the risk that the target task merely reflects a single-location artifact. Additional dataset-flow and auscultation-location summaries are provided in the supplementary material.

### 3.2 Teacher models and preliminary sanity checks

The external teacher models were strong enough to justify testing them as auxiliary guides, but they also suggested that transfer into the mixed target domain might be limited. The PhysioNet teacher showed balanced discrimination and acceptable calibration, whereas the ICBHI teacher achieved high ranking performance but a more asymmetric operating profile at the default threshold. These results support using the teachers as candidate sources of guidance, not as evidence that such guidance would necessarily transfer cleanly to the mixed task.

Preliminary fold-0 baselines further indicated that the target problem was learnable. A hand-crafted SVM reference achieved heart-task AUROC 0.8333, and a random-forest lung reference achieved AUROC 1.000 on fold 0. These exploratory models were not treated as formal competitors in the main comparison because a matched five-fold campaign was not completed for all feature-based baselines. They are therefore reported only as contextual reference points showing that the processed inputs retained usable task information.

### 3.3 Main nested five-fold comparison

**Fig 2:**
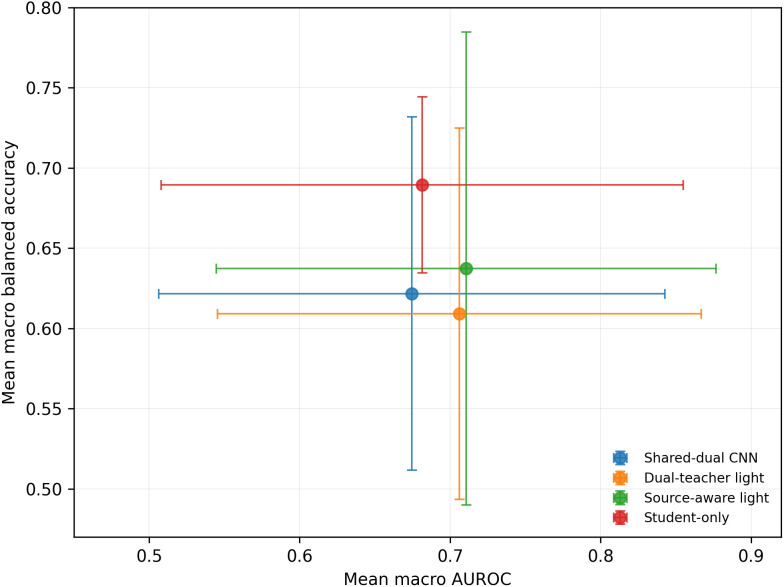
Cross-validated discrimination-versus-decision summary of the four principal calibrated models under the nested evaluation.

The main nested cross-validation comparison is summarized in Table 1, while the overall discrimination-versus-decision pattern is shown in Fig 2. After applying the same inner-validation calibration and threshold-selection procedure to all principal models, the light source-aware model achieved the highest mean discrimination, with macro AUROC 0.7107 ± 0.1659 and macro AUPRC 0.9318 ± 0.0423. The light dual-teacher model followed closely on ranking metrics, whereas the shared-dual CNN and student-only model remained lower on mean macro AUROC. Because the no-skill macro AUPRC baseline was already high (0.8586 ± 0.0225), the absolute AUPRC values should be read together with their modest margins above this baseline.

**Table 1:** Nested grouped five-fold results after inner-validation calibration and threshold selection (mean ± standard deviation).

Model	Macro AUROC	Macro bal. acc.	Macro AUPRC	Macro AUPRC baseline	Heart ECE	Lung ECE	Heart Brier	Lung Brier
Shared-dual CNN	0.6746 ± 0.1681	0.6217 ± 0.1101	0.9228 ± 0.0409	0.8586 ± 0.0225	0.1194 ± 0.0919	0.3192 ± 0.2001	0.1061 ± 0.0446	0.2527 ± 0.1555
Dual-teacher light	0.7062 ± 0.1607	0.6092 ± 0.1157	0.9302 ± 0.0400	0.8586 ± 0.0225	0.1082 ± 0.0811	0.3128 ± 0.1670	0.0946 ± 0.0361	0.2438 ± 0.1216
Source-aware light	0.7107 ± 0.1659	0.6373 ± 0.1474	0.9318 ± 0.0423	0.8586 ± 0.0225	0.1026 ± 0.0769	0.2808 ± 0.1561	0.0915 ± 0.0360	0.2222 ± 0.1150
Student-only	0.6814 ± 0.1735	0.6894 ± 0.0548	0.9252 ± 0.0477	0.8586 ± 0.0225	0.1609 ± 0.1761	0.1784 ± 0.0187	0.1340 ± 0.1024	0.1294 ± 0.0168

Once decision thresholds were considered, the ordering changed. After inner-validation temperature scaling and threshold selection, the calibrated student-only model achieved the highest mean macro balanced accuracy, 0.6894 ± 0.0548. The corresponding values were 0.6373 ± 0.1474 for the light source-aware model, 0.6217 ± 0.1101 for the shared-dual CNN, and 0.6092 ± 0.1157 for the light dual-teacher model. Thus, better ranking performance did not automatically translate into the most favorable threshold-dependent behavior.

The main comparison is therefore best read as a separation between discrimination and operating-point behavior rather than as a single model ranking. On average, the light source-aware model separated recordings most effectively, whereas the calibrated student-only model produced the most balanced thresholded decisions under the revised nested procedure. The standard deviations across folds were large relative to the mean differences between several models, so these differences should be interpreted as tendencies rather than definitive superiority.

### 3.4 Effect of calibration and threshold optimization

The main effect of calibration was not a change in AUROC, which would not be expected, but a change in how models behaved once decisions had to be thresholded. In the revised analysis, temperature scaling and threshold selection were performed only on the inner validation split of each outer fold and then applied to the outer test fold. This design gives a more conservative estimate of operating-point behavior than the previous within-fold thresholding summary.

The calibrated student-only model provides the clearest example of this separation. It did not have the highest mean macro AUROC, but it achieved the highest mean macro balanced accuracy after inner-validation threshold selection. Conversely, the light source-aware model produced the strongest ranking tendency but a lower threshold-dependent balanced accuracy. For that reason, calibration is treated here as part of the evaluated decision pipeline, and the resulting operating-point summaries are interpreted alongside fold-level variability rather than as fixed-threshold clinical performance.

**Fig 3:**
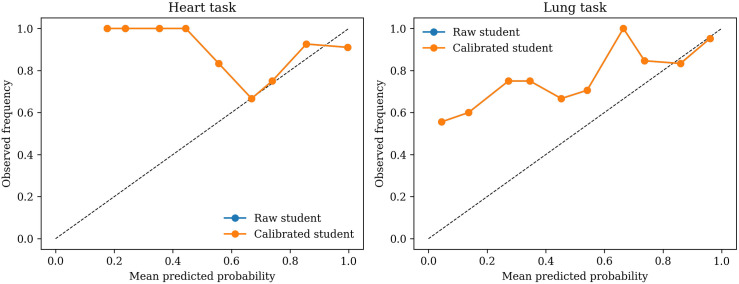
Reliability diagrams and pooled probability distributions before and after calibration.

Fig 3 summarizes the calibration curves and pooled probability distributions used to examine post-calibration operating behavior.

### 3.5 Effect of teacher guidance and source-aware weighting

The ablation results should be read cautiously because they come from fold 0 rather than the full five-fold campaign. Their role is explanatory rather than confirmatory. On fold 0, the student-only model reached macro AUROC 0.9619. The heavier dual-teacher and source-aware variants lowered this value to 0.9426 and 0.9301, respectively, even though their auxiliary objectives remained active throughout training.

Reducing the auxiliary weight improved that fold-level behavior. The light dual-teacher variant produced more balanced decisions in the exploratory fold-0 analysis, and the light source-aware variant later achieved the strongest mean discrimination in the nested five-fold comparison. That advantage was not present for every endpoint, so the safest reading is that weak external guidance may help ranking performance on average when it remains secondary to target-domain evidence, not that it provides a uniform decision-level gain.

### 3.6 Task-level interpretation

Task-level results showed that the two branches behaved differently. Lung AUROC tended to be higher than heart AUROC across the principal models, whereas the heart task remained more variable and often limited the macro discrimination summary. This pattern is consistent with the high heart-abnormal prevalence and the small number of heart-normal examples in each outer fold.

Calibration told a different part of the story. Lung ECE was higher than heart ECE for all principal models in Table 1, even though the most visible thresholding failures often appeared in the heart branch. This suggests that calibration difficulty and thresholded specificity collapse were not identical phenomena: the heart branch drove many operating-point instabilities, while the lung branch still showed less well-calibrated probability estimates under the mixed-source setting.

### 3.7 Operating-point stability across folds

The fold-wise threshold plots add information that the ranking metrics do not provide. Thresholds selected on the inner validation splits varied substantially across outer folds, particularly for the heart branch. This variation is consistent with the small validation partitions and helps explain why decision-level performance could shift even when AUROC changed less dramatically.

These threshold trajectories argue against reporting a single default cutoff without also stating how it was obtained. In the present data, workable operating points existed, but they were model- and fold-dependent rather than fixed properties of the task. The fold-by-fold supplementary table was therefore retained to show whether model ordering was stable or driven by a small number of favorable folds.

## 4 Discussion

The revised nested benchmark does not show that stronger guidance is better by default. The light source-aware model gave the best average discrimination across folds, whereas the calibrated student-only model gave the best average balanced accuracy after inner-validation threshold selection. The light dual-teacher model remained close to the source-aware model on ranking metrics but did not improve decision-level balanced accuracy, and no single model led every endpoint.

A plausible explanation is that supervision learned from single-source corpora may not transfer cleanly into a small mixed-sound task unless its influence is kept modest. PhysioNet/CinC 2016, ICBHI 2017, and HLS-CMDS differ in recording setup, source composition, and label definition. Under that mismatch, strong auxiliary losses may pull the student toward regularities that are only partly aligned with the mixed target labels. The present data are consistent with that interpretation, although the ablation evidence remains exploratory and does not establish the mechanism on its own.

The evaluation results also make clear that AUROC was not enough for this dataset. Models with stronger ranking ability could behave less favorably once thresholds were applied, and models with more balanced thresholded decisions did not necessarily have the highest AUROC. The calibrated student-only model is the clearest example: it achieved the best mean macro balanced accuracy despite lower mean discrimination than the light source-aware model. A comparison based only on discrimination would therefore have hidden a practically important part of the result.

The shared-dual CNN baseline is still informative. A shared-representation multitask design was reasonable and remained competitive, but in this dataset it did not recover the ranking behavior of the light source-aware model or the threshold-dependent behavior of the calibrated student-only model. That pattern suggests that the harder issue here was not generic parameter sharing alone; it was how to use paired source information without making decision behavior more fragile. Relative to prior comparisons built around isolated heart or lung recordings, the present benchmark is narrower but also more demanding, because both clinical decisions must be inferred from the same mixed recording.

Several limitations remain important. First, the paired mixed subset is small, and fold-wise variability is substantial, especially for the heart branch. Second, HLS-CMDS is a manikin-based corpus, so the study should be read as a controlled methodological comparison rather than as evidence of immediate bedside readiness. Third, balanced accuracy was still obtained after inner-validation threshold selection and should not be interpreted as locked-threshold external clinical performance. Fourth, the cross-validation summaries are descriptive rather than inferential, because the fold count is small and the folds are not statistically independent replicates. Fifth, the exploratory feature-based references and fold-0 ablations help interpret the results, but they do not carry the same evidentiary weight as the matched nested five-fold comparison of the principal models.

These constraints also indicate where stronger evidence would come from. Repeated nested cross-validation or a larger paired corpus would help determine whether the observed ranking and operating-point tendencies are stable. External validation on human mixed recordings is also needed, because domain shift may be larger there than in the present manikin-based setting. For the heart branch in particular, calibration-aware training or uncertainty-sensitive thresholding may prove more useful than heavier auxiliary supervision.

The gap between the best discriminator and the best decision-level baseline matters because real use requires a threshold, not only a ranking. In biomedical machine learning, the model that orders cases best is not always the model that is easiest to defend once a binary decision has to be made. The present study makes that tension visible in a small mixed-auscultation setting.

Overall, the most defensible practical reading is modest. In small paired mixed-sound settings similar to HLS-CMDS, light source-aware guidance can improve average discrimination, but a simpler target-domain model may remain difficult to surpass once calibration and threshold selection are handled carefully. The results therefore argue against assuming that additional architectural complexity will automatically improve decision quality.

## 5 Conclusions

We presented a comparative study of mixed cardiopulmonary sound classification on a low-resource paired dataset using nested grouped five-fold cross-validation. In the principal comparison, the light source-aware model achieved the best mean discrimination, whereas the calibrated student-only model achieved the best mean decision-level balanced accuracy after inner-validation temperature scaling and threshold selection. The overall pattern was a trade-off across endpoints rather than a universally superior model.

More broadly, the study suggests that mixed auscultation tasks do not necessarily benefit from stronger teacher transfer or heavier source constraints. In this paired target domain, restrained guidance was more effective for discrimination than heavy guidance, while calibration and threshold selection materially affected practical behavior. Whether the same pattern holds under larger samples and genuinely external clinical validation remains unresolved.

## Supporting information

S1 Appendix Detailed preprocessing, model settings, software environment notes, and fold-level results. (DOCX)

S1 Code Analysis scripts, model definitions, training utilities, and parameter settings used to generate the reported experiments. (ZIP)

S2 Data Dataset split tables and harmonized metadata for the target and teacher datasets. (ZIP)

S3 Data Fold-level predictions, metrics, and aggregated summary reports supporting the manuscript tables and figures. (ZIP)

S4 Checklist Completed STARD-AI checklist. (DOCX)
