# Supplementary material for: Comparative modeling of mixed cardiopulmonary sounds in a low-resource paired dataset: Discrimination, calibration, and operating-point behavior
Source: PLoS One. 2026 Jun 22;21(6):e0352180. doi: 10.1371/journal.pone.0352180 (PMC13286179; doi:10.1371/journal.pone.0352180)
Supplement: S1 Appendix — (DOCX) [file pone.0352180.s001.docx]

S1 Appendix

Revised supplementary methods, nested evaluation outputs, and fold-level results

Manuscript ID: PONE-D-26-15185

This revised appendix replaces the original fold-level results with the nested grouped five-fold revision analysis. The outer grouped folds were preserved from the submitted study, while early stopping, temperature scaling, and task-specific threshold selection were moved to an inner validation split within each outer training fold. The outer fold was reserved for final evaluation only.

# A1. Reproducibility and revision note

The revised analysis was performed after reviewer feedback identified that the original held-out fold had been used both for model selection and for final decision-level reporting. In the revised pipeline, each outer training fold was divided into an inner training subset and an inner validation subset. The inner validation subset was used for checkpoint selection, temperature scaling, and heart/lung threshold selection; the corresponding outer fold was used only for final evaluation.

The revised outputs used to update the main manuscript were: revision_nested_summary.csv, revision_fold_by_fold_supplement.csv, and revision_nested_verification_report.md. All four principal models were evaluated with the same post-hoc calibration and threshold-selection procedure.

# A2. Dataset and split audit summary

The target-domain analysis used 145 pair-level mixed cardiopulmonary triplets. Grouped five-fold cross-validation was retained, with pair_id used as the grouping variable. Each outer fold contained 29 outer-test samples. The verification report confirmed no group overlap between inner training, inner validation, and outer-test partitions.

Table A1. Summary of the revised nested evaluation protocol.

| **Protocol element** | **Revised implementation** |
| --- | --- |
| Outer evaluation | Original grouped five-fold cross-validation retained. |
| Grouping variable | pair_id / group_id; no overlap across evaluation partitions. |
| Inner validation | Created within each outer training fold. |
| Model selection | Early stopping and best checkpoint selection used the inner validation subset only. |
| Calibration | Temperature scaling was fitted on inner validation predictions only. |
| Threshold selection | Heart and lung thresholds were selected on inner validation calibrated probabilities. |
| Final evaluation | Outer fold predictions were evaluated once after applying the inner-validation temperature and thresholds. |
| Models calibrated | Shared-dual CNN, dual-teacher light, source-aware light, and student-only. |
| Teacher checkpoints | Fold-specific teacher checkpoints were used for each outer fold; no fixed fold0 fallback was used. |

# A3. Revised model-level summary

Table A2 reports the mean ± SD across the five grouped outer folds under the revised nested evaluation. AUPRC values are reported together with the prevalence-defined no-skill AUPRC baseline.

| **Model** | **Macro AUROC** | **Macro AUPRC** | **Macro AUPRC baseline** | **Macro balanced accuracy** | **Heart ECE** | **Lung ECE** | **Heart Brier** | **Lung Brier** |
| --- | --- | --- | --- | --- | --- | --- | --- | --- |
| Shared-dual CNN | 0.6746 ± 0.1681 | 0.9228 ± 0.0409 | 0.8586 ± 0.0225 | 0.6217 ± 0.1101 | 0.1194 ± 0.0919 | 0.3192 ± 0.2001 | 0.1061 ± 0.0446 | 0.2527 ± 0.1555 |
| Dual-teacher light | 0.7062 ± 0.1607 | 0.9302 ± 0.0400 | 0.8586 ± 0.0225 | 0.6092 ± 0.1157 | 0.1082 ± 0.0811 | 0.3128 ± 0.1670 | 0.0946 ± 0.0361 | 0.2438 ± 0.1216 |
| Source-aware light | 0.7107 ± 0.1659 | 0.9318 ± 0.0423 | 0.8586 ± 0.0225 | 0.6373 ± 0.1474 | 0.1026 ± 0.0769 | 0.2808 ± 0.1561 | 0.0915 ± 0.0360 | 0.2222 ± 0.1150 |
| Student-only | 0.6814 ± 0.1735 | 0.9252 ± 0.0477 | 0.8586 ± 0.0225 | 0.6894 ± 0.0548 | 0.1609 ± 0.1761 | 0.1784 ± 0.0187 | 0.1340 ± 0.1024 | 0.1294 ± 0.0168 |

# A4. Fold-level supplementary results

Table A3 provides fold-by-fold macro-level results for all four calibrated principal models. The full task-level outputs, thresholds, temperatures, sensitivities and specificities are supplied in revision_fold_by_fold_supplement.csv.

| **Model** | **Fold** | **n** | **Heart prev.** | **Lung prev.** | **Macro AUROC** | **Macro AUPRC** | **AUPRC baseline** | **Macro balanced accuracy** | **Heart ECE** | **Lung ECE** |
| --- | --- | --- | --- | --- | --- | --- | --- | --- | --- | --- |
| Shared-dual CNN | 0 | 29 | 0.897 | 0.828 | 0.816 | 0.957 | 0.862 | 0.667 | 0.084 | 0.312 |
| Shared-dual CNN | 1 | 29 | 0.897 | 0.793 | 0.674 | 0.917 | 0.845 | 0.660 | 0.188 | 0.106 |
| Shared-dual CNN | 2 | 29 | 0.931 | 0.828 | 0.424 | 0.883 | 0.879 | 0.581 | 0.046 | 0.605 |
| Shared-dual CNN | 3 | 29 | 0.931 | 0.828 | 0.839 | 0.972 | 0.879 | 0.746 | 0.036 | 0.410 |
| Shared-dual CNN | 4 | 29 | 0.897 | 0.759 | 0.619 | 0.884 | 0.828 | 0.455 | 0.244 | 0.163 |
| Dual-teacher light | 0 | 29 | 0.897 | 0.828 | 0.955 | 0.994 | 0.862 | 0.719 | 0.089 | 0.145 |
| Dual-teacher light | 1 | 29 | 0.897 | 0.793 | 0.738 | 0.910 | 0.845 | 0.617 | 0.042 | 0.558 |
| Dual-teacher light | 2 | 29 | 0.931 | 0.828 | 0.529 | 0.898 | 0.879 | 0.508 | 0.069 | 0.289 |
| Dual-teacher light | 3 | 29 | 0.931 | 0.828 | 0.695 | 0.946 | 0.879 | 0.725 | 0.092 | 0.389 |
| Dual-teacher light | 4 | 29 | 0.897 | 0.759 | 0.613 | 0.904 | 0.828 | 0.476 | 0.249 | 0.184 |
| Source-aware light | 0 | 29 | 0.897 | 0.828 | 0.964 | 0.995 | 0.862 | 0.823 | 0.091 | 0.112 |
| Source-aware light | 1 | 29 | 0.897 | 0.793 | 0.740 | 0.908 | 0.845 | 0.701 | 0.051 | 0.400 |
| Source-aware light | 2 | 29 | 0.931 | 0.828 | 0.529 | 0.898 | 0.879 | 0.508 | 0.069 | 0.227 |
| Source-aware light | 3 | 29 | 0.931 | 0.828 | 0.720 | 0.955 | 0.879 | 0.688 | 0.064 | 0.485 |
| Source-aware light | 4 | 29 | 0.897 | 0.759 | 0.600 | 0.903 | 0.828 | 0.466 | 0.238 | 0.181 |
| Student-only | 0 | 29 | 0.897 | 0.828 | 0.712 | 0.943 | 0.862 | 0.636 | 0.464 | 0.164 |
| Student-only | 1 | 29 | 0.897 | 0.793 | 0.654 | 0.879 | 0.845 | 0.683 | 0.017 | 0.196 |
| Student-only | 2 | 29 | 0.931 | 0.828 | 0.415 | 0.870 | 0.879 | 0.640 | 0.150 | 0.189 |
| Student-only | 3 | 29 | 0.931 | 0.828 | 0.732 | 0.956 | 0.879 | 0.724 | 0.104 | 0.191 |
| Student-only | 4 | 29 | 0.897 | 0.759 | 0.894 | 0.977 | 0.828 | 0.763 | 0.070 | 0.153 |

# A5. Temperature and threshold audit

Table A4 summarizes the learned task-specific temperatures and thresholds across folds. These values were estimated on the inner validation split and then applied to the outer-test fold.

| **Model** | **Heart temperature** | **Lung temperature** | **Heart threshold** | **Lung threshold** |
| --- | --- | --- | --- | --- |
| Shared-dual CNN | 0.7609 ± 0.3157 | 1.4294 ± 0.7187 | 0.8318 ± 0.1138 | 0.4668 ± 0.2212 |
| Dual-teacher light | 0.8451 ± 0.7845 | 1.3328 ± 1.0333 | 0.6877 ± 0.3772 | 0.4113 ± 0.1800 |
| Source-aware light | 0.9554 ± 0.8586 | 1.3020 ± 0.8740 | 0.6999 ± 0.3839 | 0.4558 ± 0.1739 |
| Student-only | 1.2927 ± 0.8523 | 1.5319 ± 0.5701 | 0.6600 ± 0.2995 | 0.7668 ± 0.1248 |

# A6. Verification report

The final verification script passed for all 20 model-fold calibration outputs. It confirmed that all calibrated metrics JSON files contained the required AUPRC baseline, temperature, threshold, and split-audit fields; that calibration_split was inner_val; that evaluation_split was outer_test; and that group overlap between calibration and outer evaluation partitions was zero.

The revised results should therefore be read as a more conservative estimate of model behavior than the original within-fold threshold-selection summary. The relatively large fold-to-fold standard deviations are retained in the supplementary table to make this uncertainty visible.

# A7. Supporting data files included with the revision package

revision_nested_summary.csv

revision_nested_summary.md

revision_fold_by_fold_supplement.csv

revision_nested_verification_report.md

revision_runbook.md
