# Supplementary material for: Comparative modeling of mixed cardiopulmonary sounds in a low-resource paired dataset: Discrimination, calibration, and operating-point behavior
Source: PLoS One. 2026 Jun 22;21(6):e0352180. doi: 10.1371/journal.pone.0352180 (PMC13286179; doi:10.1371/journal.pone.0352180)
Supplement: S4 Checklist — (DOCX) [file pone.0352180.s005.docx]

S4 Checklist. Completed STARD-AI reporting checklist

This checklist maps the revised manuscript to the STARD-AI reporting items for artificial-intelligence-centered diagnostic accuracy studies. Locations are given by manuscript section because exact page and line numbers may shift during editorial typesetting.

Checklist source: Sounderajah et al., The STARD-AI reporting guideline for diagnostic accuracy studies using artificial intelligence, Nature Medicine, 2025. The downloadable STARD-AI checklist contains 40 reporting items, including AI-specific dataset, model-evaluation, and fairness considerations.

| **Section/topic** | **No.** | **STARD-AI item** | **Reported location** | **Author comment** |
| --- | --- | --- | --- | --- |
| Title or abstract | 1 | Identification as a study reporting AI-centred diagnostic accuracy and reporting at least one measure of accuracy within the title or abstract. | Title; Abstract | Reported in title and abstract through automated mixed cardiopulmonary sound classification and accuracy metrics. |
| Abstract | 2 | Structured summary of study design, methods, results and conclusions. | Abstract | The abstract summarizes dataset, models, nested evaluation, calibration and principal results. |
| Introduction | 3 | Scientific and clinical background, intended use of the index test, and integration into workflow, if applicable. | Introduction | The manuscript frames low-resource paired cardiopulmonary sound classification as a comparative modeling problem. |
| Introduction | 4 | Study objectives and hypotheses. | End of Introduction | Objectives are comparative discrimination, calibration and operating-point behavior under mixed cardiopulmonary recordings. |
| Methods - Study design | 5 | Whether data collection was planned before the index test and reference standard were performed or after them. | Methods 2.1 | Retrospective secondary analysis of an available paired dataset. |
| Methods - Ethics | 6 | Formal ethics approval; if not required, justify why. | Ethics statement / Methods | The manuscript states the use of de-identified/public or secondary data as applicable. |
| Methods - Participants | 7 | Eligibility criteria, with separate inclusion and exclusion criteria in applied order at participant and data level. | Methods 2.1 | Triplet construction and exclusion criteria are described. |
| Methods - Participants | 8 | Basis on which potentially eligible participants were identified. | Methods 2.1 | Eligible samples were identified from the paired HLS-CMDS mixed subset. |
| Methods - Participants | 9 | Where and when potentially eligible participants were identified. | Methods 2.1 | Dataset source and setting are reported; dates are limited by the source dataset documentation. |
| Methods - Participants | 10 | Whether participants formed a consecutive, random, or convenience series. | Methods 2.1 / Limitations | The analysis uses available paired recordings and is treated as low-resource convenience data. |
| Methods - Dataset | 11 | Source of the data and whether it was routinely collected, specifically collected, or open-source. | Methods 2.1; Data availability | The data source and paired mixed subset are described. |
| Methods - Dataset | 12 | Who undertook annotations and how, if applicable. | Methods 2.1 | Label provenance follows the source dataset; no new clinical annotation was introduced. |
| Methods - Dataset | 13 | Devices used to capture data; software versions used to engineer the index test. | Methods 2.1; Methods 2.7 | Recording and software notes are provided to the extent available from the source dataset; Python analysis stack is described. |
| Methods - Dataset | 14 | Data acquisition protocols and preprocessing details sufficient for replication. | Methods 2.2; S1 Appendix | Preprocessing, clip duration, sample rate, and feature/model settings are reported. |
| Methods - Test methods | 15a | Index test in sufficient detail to allow replication. | Methods 2.3-2.5 | Model families and inference outputs are described. |
| Methods - Test methods | 15b | How the index test was developed, including training, validation, testing and external evaluation with sample sizes. | Methods 2.6; S1 Appendix | Nested grouped five-fold training, calibration and final outer evaluation are described. |
| Methods - Test methods | 15c | Definition and rationale for test positivity cut-offs or result categories, distinguishing pre-specified from exploratory. | Methods 2.6 | Thresholds were selected on inner validation splits using balanced accuracy and applied to outer folds. |
| Methods - Test methods | 15d | Specified end user and level of expertise required. | Discussion / Limitations | The system is positioned as a benchmark/comparative research tool rather than a deployed clinical device. |
| Methods - Reference standard | 16a | Reference standard in sufficient detail to allow replication. | Methods 2.1 | Binary abnormal labels for heart and lung tasks are defined from the dataset labels. |
| Methods - Reference standard | 16b | Rationale for choosing the reference standard. | Methods 2.1 | The study uses the dataset-provided abnormality labels as reference outcomes. |
| Methods - Reference standard | 16c | Definition and rationale for reference-standard positivity cut-offs/categories. | Methods 2.1 | Heart and lung abnormal labels are treated as the target conditions. |
| Methods - Blinding | 17a | Whether clinical information and reference standard results were available to performers/readers of the index test. | Methods 2.6 | Models received acoustic-derived inputs; evaluation labels were not used during outer-test inference. |
| Methods - Blinding | 17b | Whether clinical information and index test results were available to assessors of the reference standard. | Methods 2.1 | Reference labels came from the existing dataset, not from model outputs. |
| Methods - Analysis | 18 | Methods for estimating or comparing diagnostic accuracy. | Methods 2.6-2.7 | AUROC, AUPRC, prevalence-defined AUPRC baseline, ECE, Brier score, sensitivity, specificity and balanced accuracy are reported. |
| Methods - Analysis | 19 | Handling of indeterminate index test or reference standard results. | Methods 2.1 / Limitations | Samples without usable paired labels or triplet construction were excluded. |
| Methods - Analysis | 20 | Handling of missing data on index test and reference standard. | Methods 2.1 / S1 Appendix | Available complete triplets were analyzed; exclusions are described. |
| Methods - Analysis | 21 | Analyses of variability in diagnostic accuracy. | Results; S1 Appendix | Mean ± SD across five grouped outer folds and fold-by-fold supplementary results are provided. |
| Methods - Analysis | 22 | Intended sample size and how determined. | Methods 2.1 / Limitations | No formal prospective sample-size calculation; the study uses all available low-resource paired triplets. |
| Methods - Analysis | 23 | Performance error analysis and algorithmic bias/fairness assessments if undertaken. | Results; Discussion | Calibration, threshold behavior and fold variability are analyzed; fairness analysis is limited by available metadata. |
| Results - Participants/dataset | 24 | Flow of participants, using a diagram. | Methods / S1 Appendix | Dataset filtering and the final 145 triplets are reported; no clinical participant flow diagram was added because the study is secondary data analysis. |
| Results - Participants/dataset | 25 | Baseline demographic, clinical and technical characteristics of training, validation and test sets, if applicable. | Methods 2.1; S1 Appendix | Available technical characteristics and split composition are reported. |
| Results - Participants/dataset | 26a | Distribution of severity of disease in those with target condition. | Results / Limitations | Severity distributions were not available beyond abnormality labels. |
| Results - Participants/dataset | 26b | Distribution of alternative diagnoses in those without target condition. | Results / Limitations | Alternative-diagnosis detail was not available for the target subset. |
| Results - Participants/dataset | 27 | Time interval and interventions between index test and reference standard. | Not applicable | The study used paired recordings and existing labels rather than a prospective clinical workflow. |
| Results - Participants/dataset | 28 | Whether datasets represent the distribution expected from intended-use population. | Discussion / Limitations | Generalizability is discussed cautiously because the dataset is small and paired. |
| Results - Participants/dataset | 29 | For external evaluation, assessment of how it differs from training, validation and test sets. | Methods / Discussion | External teacher datasets are described; final target-domain evaluation uses nested grouped folds. |
| Results - Test results | 30 | Cross tabulation of index test results by reference standard results or their distribution. | Results; S1 Appendix | Sensitivity, specificity, thresholds and fold-level outputs are supplied in supplementary CSV outputs. |
| Results - Test results | 31 | Estimates of diagnostic accuracy and precision. | Results; Table 1; S1 Appendix | Mean ± SD across folds are reported for all principal metrics. |
| Results - Test results | 32 | Adverse events from performing the index test or reference standard. | Not applicable | This was an offline secondary analysis of recordings; no study intervention was performed. |
| Discussion | 33 | Study limitations, including sources of potential bias, statistical uncertainty and generalisability. | Discussion | Low sample size, fold variability, calibration uncertainty and generalizability are discussed. |
| Discussion | 34 | Implications for practice, including intended use and clinical role. | Discussion | The paper avoids deployment claims and frames the work as a benchmark for low-resource mixed cardiopulmonary sound modeling. |
| Discussion | 35 | Ethical considerations and adherence to ethical standards associated with index test and fairness. | Discussion / Ethics | Ethics and fairness limitations are acknowledged; no clinical deployment is claimed. |
| Other information | 36 | Registration number and name of registry. | Not applicable | No prospective diagnostic accuracy trial registration was used for this retrospective computational study. |
| Other information | 37 | Where the full study protocol can be accessed. | Data/code availability; S1 Code | The analysis protocol is represented by the accompanying code and runbook. |
| Other information | 38 | Sources of funding and other support; role of funders. | Funding statement | Funding/support statement is reported in the manuscript. |
| Other information | 39 | Commercial interests, if applicable. | Competing interests statement | Competing interests are stated in the manuscript. |
| Other information | 40a | Availability of datasets and code, including restrictions on reuse and repurposing. | Data availability; Supporting information | Dataset/code availability and restrictions are stated. |
| Other information | 40b | Whether outputs are stored, auditable and available for evaluation, if necessary. | S1 Appendix; S3 Data | Revision outputs include summary, fold-level supplement and verification report. |
